# Re-Use of Polycarbonate from Compact Discs to Enhance the Thermal Stability of Polylactic Acid Blends

**DOI:** 10.3390/polym17050606

**Published:** 2025-02-24

**Authors:** Samaneh Dehghani, Dutchanee Pholharn, Yottha Srithep

**Affiliations:** 1 Manufacturing and Materials Research Unit, Department of Manufacturing Engineering, Faculty of Engineering, Mahasarakham University, Mahasarakham 44150, Thailand; samane.dehghani@gmail.com; 2 Department of Rubber and Polymer Technology, Faculty of Science and Technology, Rajabhat Mahasarakham University, Mahasarakham 44000, Thailand; dutchanee.ph@rmu.ac.th

**Keywords:** polylactic acid, recycled polycarbonate, thermal stability, crystallinity, tensile toughness

## Abstract

This study investigates enhancing polylactic acid (PLA) by incorporating recycled polycarbonate (r-PC) to address PLA’s inherent brittleness and limited thermal stability. Blends with varying PLA/r-PC ratios (100:0 to 0:100) were prepared using an internal mixer, with r-PC sourced from discarded compact discs. The thermogravimetric analysis (-A) demonstrated significant improvements in the thermal stability. The degradation onset temperature (T_5_ wt%) increased from approximately 315 °C for pure PLA to about 400 °C in the blends, with a maximum decomposition temperature (T_max_) of 520 °C observed for pure r-PC. The char residue also increased markedly, from 1.35% in pure PLA to 24.42% in r-PC, indicating enhanced thermal resistance. Differential scanning calorimetry (DSC) revealed a considerable reduction in PLA crystallinity, declining from 68.17% in pure PLA to 10.32% in the 10PLA90r-PC blend, indicative of the disruption of PLA’s crystalline structure. The X-ray diffraction (XRD) analysis supported these findings, showing a transition to a predominantly amorphous structure at higher r-PC contents. Tensile testing highlighted the mechanical improvements achieved through blending. While pure PLA exhibited brittle failure, the 30PLA70r-PC blend displayed plastic deformation, signifying improved toughness. The stress–strain analysis revealed that the 30PLA70r-PC blend achieved a peak toughness of 8725 kJ/m^3^, nearly ten times higher than the 924 kJ/m^3^ recorded for pure PLA. However, excessive r-PC content introduced brittleness, diminishing toughness. The dynamic mechanical thermal analysis (DMTA) demonstrated a broadening of the glass transition range, with the T_g_ shifting from 61 °C for pure PLA to 141 °C in r-PC-dominant blends, reflecting improved phase interactions between the two polymers. Scanning electron microscopy (SEM) revealed significant morphological changes; at high r-PC contents, phase separation and voids were observed, leading to reduced mechanical performance. These results highlight the synergistic potential of blending PLA’s biodegradability with r-PC’s superior thermal and mechanical properties.

## 1. Introduction

Using biodegradable polymers like polylactic acid (PLA), the development of sustainable materials has expanded significantly, driven by growing environmental awareness and the increasing demand for eco-friendly alternatives. PLA, derived from renewable resources such as corn starch and sugarcane, has observed remarkable growth in production and applications over the past decade [1,2,3,4]. By 2022, the global PLA market had surpassed USD 3 billion, driven by its extensive use in packaging, medical devices, and 3D printing. PLA is widely valued for its biocompatibility, biodegradability, and reduced environmental impact. However, its brittleness, low thermal resistance, and limited mechanical strength pose significant challenges, restricting its use in high-performance applications. These limitations underscore the urgent need for innovative strategies that combine sustainability with enhanced material properties [5,6,7,8,9].

To address PLA’s weaknesses, researchers have explored blending it with various additives and polymers to improve its thermal and mechanical performance. For instance, polyhydroxyalkanoates (PHAs), a class of biodegradable polyesters, have been blended with PLA to improve flexibility and reduce brittleness [10,11,12]. Similarly, thermoplastic starch (TPS) has been used to enhance PLA’s biodegradability [13,14,15], though it often compromises thermal stability. Polybutylene adipate terephthalate (PBAT) [16,17,18], a biodegradable polymer, has been introduced to improve impact resistance, while epoxidized soybean oil (ESO) has served as a plasticizer and compatibilizer to reduce brittleness and improve toughness [19,20]. Despite these advancements, many blends still struggle to achieve a balance between improved performance and sustainability, especially in applications requiring high thermal stability and mechanical strength.

In recent years, advancements in post-consumer recycled (PCR) waste management have significantly enhanced the mechanical recycling of various thermoplastics, such as polyethylene, polypropylene, and polyamides, enabling their reuse in diverse applications [21,22,23,24]. However, engineering plastics like polycarbonate (PC) and polymethyl methacrylate (PMMA) have received comparatively less attention in recycling efforts [25,26,27,28]. Among these, polycarbonate (PC) stands out due to its exceptional impact resistance, optical clarity, and thermal stability, making it indispensable in industries such as electronics, automotive, and construction. A particularly abundant source of recyclable PC is compact discs (CDs), which are being discarded in large quantities due to the shift toward digital media. Despite its potential, the recycling rate of PC remains relatively low, highlighting the need for innovative approaches to repurpose this valuable material effectively [29,30,31]. Blending recycled PC (r-PC) with PLA offers a promising solution to address both challenges—the inherent weaknesses of PLA and the underutilization of PC in recycling. Compared to other polymers commonly blended with PLA, PC provides superior thermal stability, impact resistance, and mechanical strength, making it an excellent candidate for enhancing PLA’s properties. Moreover, the use of recycled PC from CDs aligns with the principles of a circular economy by converting post-consumer waste into high-value applications. This approach not only improves the performance of PLA but also contributes to reducing plastic waste and addressing environmental concerns [32,33,34,35].

In this study, we investigated the degradation behavior, crystallization percentage, and tensile toughness of PLA blended with recycled polycarbonate (r-PC) in various ratios. The r-PC, obtained from discarded CDs, was used to enhance the thermal stability and mechanical properties of PLA blends. To achieve this, PLA and r-PC were blended in ratios of 100:0, 90:10, 70:30, 50:50, 30:70, 10:90, and 0:100 using an internal mixer. These blends were comprehensively analyzed to evaluate their decomposition profiles, thermal properties, crystallization behavior, and mechanical toughness, and the results were compared across the different ratios. The novelty of this work lies in re-using waste polycarbonate into PLA blends, offering a sustainable approach to address PLA limitations.

## 2. Experimental Section

### 2.1. Materials and Sample Preparation

Polylactic acid (PLA), grade L175, was procured from Total Corbion Co., Ltd., Rayong, Thailand. Recycled polycarbonate (r-PC) was sourced from discarded CDs supplied by Princo Co., Ltd., Hsinchu City, Taiwan. Recycled PC preparation involved cleaning the CDs thoroughly to eliminate contaminants, followed by grinding them into flakes using a mechanical grinder. The PLA and r-PC were blended in an internal mixer (MX 500, Charoen Tut Co., Ltd., Samutprakarn, Thailand) at 240 °C for 4 min to ensure homogeneous mixing. Blends were prepared at the following PLA/r-PC ratios: 100:0, 90:10, 70:30, 50:50, 30:70, 10:90, and 0:100. All prepared blends were subsequently processed using an injection molding machine (INJ-58T, Charoen Tut Co., Ltd., Samutprakarn, Thailand) at 210 °C to produce test specimens.

### 2.2. Characterization

#### 2.2.1. Thermogravimetric Analysis (TGA)

The thermal stability and decomposition behavior of the samples were analyzed using a thermogravimetric analyzer (TGA, SDT Q600, TA Instruments, New Castle, DE, USA). Each sample was heated from 50 °C to 600 °C at a rate of 20 °C/min under a dynamic nitrogen atmosphere. This method allowed for evaluating the thermal degradation profile of PLA specimens blended with r-PC at varying ratios. The weight loss percentage was calculated during heating with: weight loss (%) = (mi − mt/mi) × 100, where mi: initial mass and mt: final mass.

#### 2.2.2. Differential Scanning Calorimetry (DSC)

The thermal properties of injection-molded samples were studied using a Perkin Elmer DSC 4000 differential scanning calorimeter (PerkinElmer, Waltham, MA, USA). Each sample was sealed in an aluminum pan and subjected to a temperature cycle involving: Heating rate: 0 °C to 250 °C at 10 °C/min. Cooling rate: 250 °C to 0 °C at 5 °C/min. Reheating rate: 0 °C to 250 °C at 10 °C/min. This analysis yielded critical thermal parameters such as glass transition temperature (T_g_), cold crystallization temperature (T_cc_), cold crystallization enthalpy (ΔH_cc_), melting temperature (T_m_), and melting enthalpy (ΔH_m_). The crystallinity percentage (X_c_) of the blends was calculated using the formula: X_c_ (%) = (ΔH_m_ − ΔH_cc_)/(ΔH_m0_ × w) × 100, where ΔH_m0_: melting enthalpy for 100% crystalline of PLA 93 J/g, w: weight fraction of PLA in the blend.

#### 2.2.3. X-Ray Diffraction (XRD) Analysis

The crystalline structure of the samples was investigated using X-ray diffraction (XRD) with a Bruker D8 Advance system (Bruker Bio Spin AG, Karlsruhe, Germany). Thin-film samples were mounted on the XRD platform and scanned in the wide-angle mode from 2° to 40° at a scanning rate of 2°/min under computer control. The crystallite size (L) was determined using the Scherrer equation:

L = K λ/β cos θ

where K is the shape factor (0.9), λ is the X-ray wavelength (Cu Kα, 0.154 nm), β is the full-width at half-maximum (FWHM) of the peak in radians, and θ is the Bragg angle. The FWHM values were extracted from the XRD peak broadening to calculate the crystallite size, providing insight into the crystalline domain structure within the polymer matrix.

#### 2.2.4. Tensile Analysis

The mechanical properties of the samples were evaluated using a tensile testing machine (NRI-TS500-2S, Narin Instrument Co., Ltd., Samutprakarn, Thailand) equipped with a 5 kN load cell. Tests were conducted at a crosshead speed of 5 mm/min, and five specimens per formulation were analyzed to ensure accurate and reproducible results. The average tensile properties were reported.

#### 2.2.5. Dynamic Mechanical Thermal Analysis (DMTA)

Dynamic mechanical thermal analysis (DMTA) was performed on injection-molded samples (dimensions: 17.6 × 12.7 × 3.2 mm3) using a TA Q800 (TA Instruments, Tokyo, Japan). The test was conducted in a single cantilever mode over a temperature range from 30 °C to 180 °C at a heating rate of 2 °C/min. A frequency of 1 Hz and a strain amplitude of 0.1% were applied to measure the storage modulus (E′), loss modulus (E″), and damping factor (tan δ). The obtained data provided information on the viscoelastic behavior and glass transition temperature of the polymer blends, allowing for an assessment of the effect of the blend composition on the mechanical performance.

#### 2.2.6. Scanning Electron Microscopy (SEM) Analysis

The surface morphology of the samples was examined using scanning electron microscopy (SEM) (HITACHI TM4000Plus, Tokyo, Japan). To ensure a clean fracture surface, samples were first immersed in liquid nitrogen and then fractured. The fractured surfaces were coated with a 20-nanometer layer of gold using a sputter coater to improve electrical conductivity before imaging. SEM micrographs were captured at various magnifications to observe the phase distribution, compatibility between polymer phases, and potential void formations at the interface.

## 3. Results and Discussion

### 3.1. Effect of r-PC Addition on Thermal Stability of PLA

**Figure 1:**
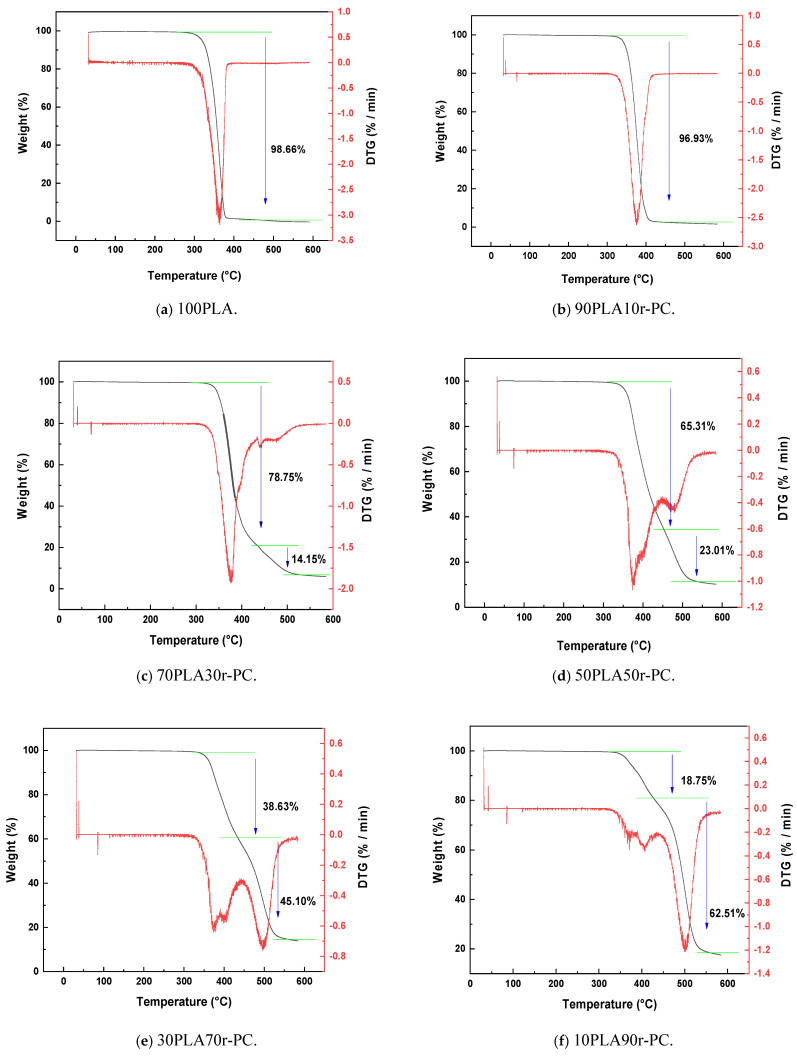

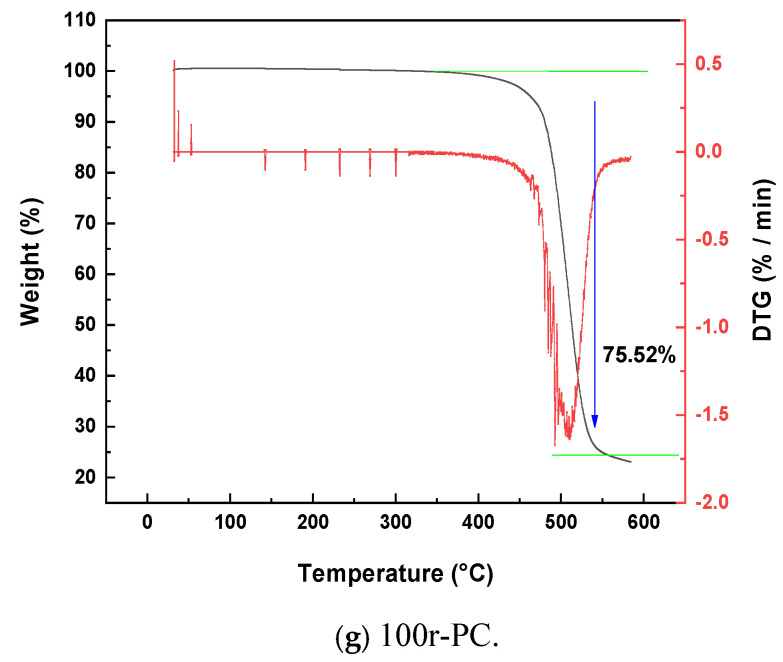
TGA and DTG thermograms of PLA/r-PC blends at various ratios: (**a**) 100PLA, (**b**) 90PLA10r-PC, (**c**) 70PLA30r-PC, (**d**) 50PLA50r-PC, (**e**) 30PLA70r-PC, (**f**) 10PLA90r-PC, and (**g**) 100r-PC. The black curve represents the TGA results, while the red curve corresponds to the DTG analysis.

The thermal stability and decomposition behavior of pure PLA (100PLA), pure recycled polycarbonate (100r-PC), and their blends were analyzed using thermogravimetric analysis (TGA). The results reveal significant changes in the degradation patterns of PLA upon adding r-PC, as shown in Figure 1. The pure PLA exhibited a single-step degradation mechanism, with an onset degradation temperature (T_d5_) of approximately 330 °C and a maximum degradation temperature (T_max_) near 370 °C. However, blending PLA with r-PC introduced a two-step degradation process, clearly influenced by the proportion of r-PC in the blend.

The two-step degradation pattern observed in PLA/r-PC blends is attributed to the degradation of individual components. The first step corresponds to PLA, which degrades at lower temperatures due to its inherently lower thermal stability [35]. The second step corresponds to r-PC, degrading at higher temperatures due to its aromatic structure and superior thermal resistance. For instance, the 70PLA30r-PC blend exhibited a weight loss of 78.74% in the first step (330–410 °C) and 14.14% in the second step (450–550 °C). The residual char content for this blend was 7.12%, with T_max_ values of ~370 °C for PLA and ~500 °C for r-PC degradation [32].

As the r-PC content increased, the thermal stability of the blends improved progressively. The 50PLA50r-PC blend displayed reduced weight loss during the first degradation step (65.3%) and an increased contribution of r-PC to the second step (23.01%). The final char residue for this composition rose to 11.69%, indicating enhanced thermal resistance due to synergistic interactions between PLA and r-PC. Blends with higher r-PC content, such as 30PLA70r-PC and 10PLA90r-PC, exhibited further improvements. The Td5 shifted to higher temperatures, and the T_max_ for the r-PC degradation step increased to ~520 °C. Notably, the 10PLA90r-PC blend retained 18.75% char residue, compared to 24.42% for pure r-PC, underscoring the thermal stability imparted by the aromatic structure of r-PC.

Adding r-PC also significantly influenced the char formation during thermal degradation. The pure PLA showed minimal char residue (1.35%), reflecting its lower thermal resistance. In contrast, r-PC-rich blends demonstrated progressively higher char residues, with the 10PLA90r-PC blend retaining 18.75% and pure r-PC retaining 24.42%. These findings align with prior studies, such as Lin et al. [32], which reported similar enhancements in thermal stability when polycarbonate was blended with PLA.

The enhanced thermal stability observed in r-PC-rich blends can be attributed to two primary factors. First, the high thermal resistance of r-PC delays heat transfer to PLA during thermal degradation. Second, potential molecular interactions between PLA and r-PC stabilize the degradation pathway. These results suggest that incorporating r-PC into PLA can significantly improve its thermal resistance, making the blend more suitable for applications requiring enhanced thermal stability.

Table 1 provides a summary of key thermal decomposition parameters, including T_d5_, T_max_ values, weight loss percentages for each degradation step, and char residue. The data clearly demonstrate the progressive improvement in thermal stability with increasing r-PC content, further validating the synergistic effects of PLA and r-PC blending [33].

**Figure 2:**
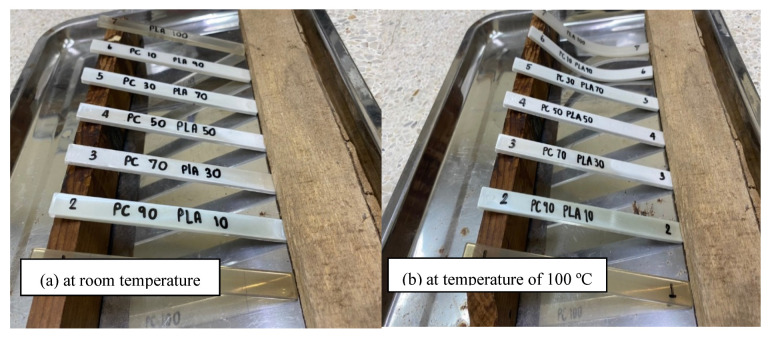
Heat resistance of the samples: (**a**) PLA/r-PC blends at room temperature; (**b**) PLA/r-PC blends after exposure to 100 °C for 30 min.

To illustrate the enhanced thermal stability of PLA through blending with r-PC, the PLA/r-PC blends were subjected to thermal exposure at 100 °C for 30 min, as depicted in Figure 2. Under these conditions, the pure PLA (Figure 2b) displayed pronounced deformation, which can be attributed to its semi-crystalline nature and relatively low glass transition temperature (T_g_) of approximately 61 °C. This low T_g_ limits the thermal resistance of pure PLA, making it susceptible to deformation at elevated temperatures.

In contrast, adding r-PC to PLA markedly reduced the deformation of the blends, particularly when the r-PC content exceeded 30%. This significant improvement in the deformation stability is primarily attributed to the higher glass transition temperature of r-PC (~141 °C), which imparts superior resistance to thermal deformation. The higher T_g_ of r-PC reinforces the blend structure at elevated temperatures, thereby enhancing the overall thermal stability of the PLA/r-PC blends [36].

### 3.2. Effect of r-PC Addition on the Crystallinity of PLA Blends

**Figure 3:**
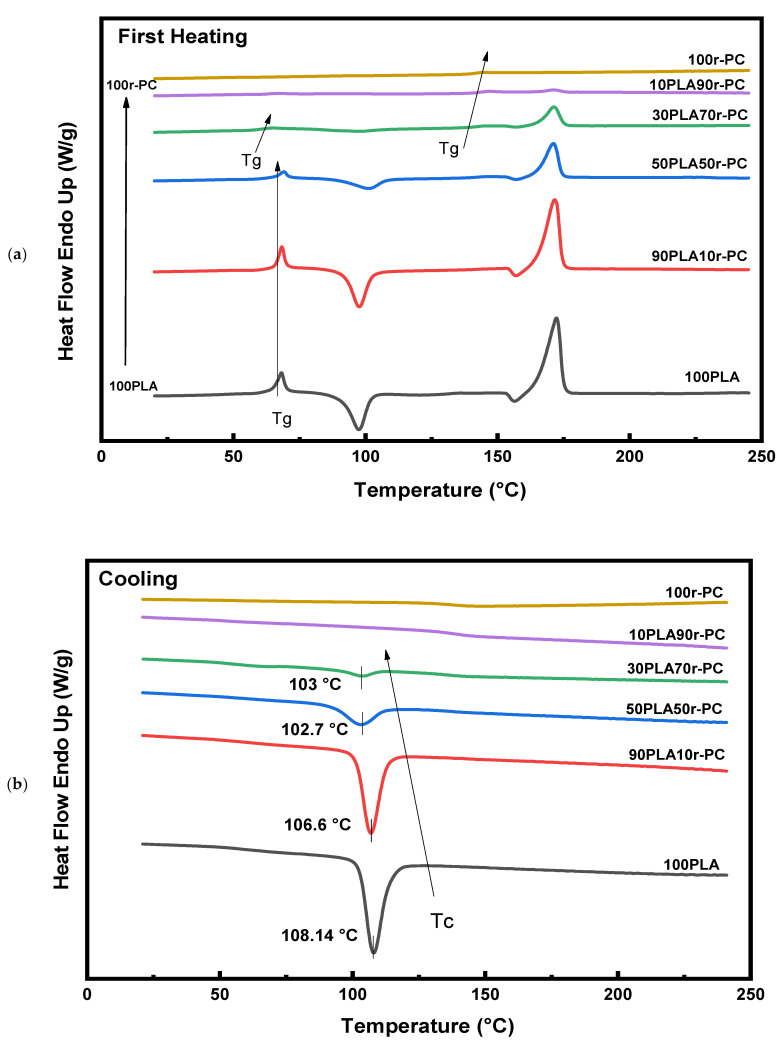

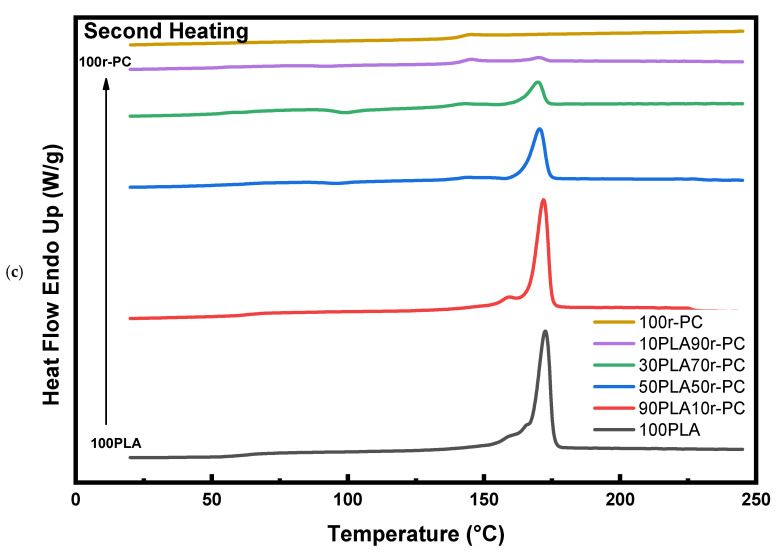
DSC thermograms of PLA/r-PC blends at various ratios of 100PLA, 90PLA10r-PC, 50PLA50r-PC, 30PLA70r-PC, 10PLA90r-PC. and 100r-PC. (**a**) First heating cycle. (**b**) Cooling cycle. (**c**) Second heating cycle.

The differential scanning calorimetry (DSC) results, as illustrated in Figure 3 and summarized in Table 2, provide critical insights into the thermal transitions and crystallinity of PLA/r-PC blends. The first heating thermograms revealed a single glass transition temperature (T_g_) for pure PLA at approximately 66 °C, along with a sharp melting peak at 172.5 °C, characteristic of its semicrystalline nature. However, as the r-PC content increased, additional thermal transitions emerged. Blends containing 50% or more r-PC exhibited a secondary T_g_ around 143–144 °C, corresponding to the amorphous nature of r-PC. The primary T_g_ of PLA showed slight shifts in the blends, suggesting molecular interactions between PLA and r-PC. The melting temperature (T_m_) of PLA decreased slightly with the increase in the r-PC content, while the intensity of the melting peaks diminished, reflecting disruptions in the PLA crystalline phase due to r-PC incorporation. These changes indicate a weakening of the ordered crystalline regions as the amorphous r-PC interferes with the regular packing of PLA chains [37].

The cooling thermograms (Figure 3b) further corroborated these findings by showing a significant reduction in crystallization temperature (T_c_) with the increase in the r-PC content. For the pure PLA, Tc was observed at 108.14 °C, but in blends with higher r-PC content, T_c_ was either significantly reduced or completely undetectable. This suggests a retardation in crystallization kinetics, likely caused by the dilution of PLA’s crystalline phase and steric hindrance imposed by r-PC’s amorphous nature. The presence of r-PC suppressed the nucleation and growth of PLA crystals, leading to a reduction in crystallinity [38].

In the second heating thermograms, a marked decline in both the melting enthalpy (ΔH_m_) and cold crystallization enthalpy (ΔH_cc_) was observed as the r-PC content increased. The crystallinity of the pure PLA, which was initially 68.17%, progressively decreased to just 10.32% in the 10PLA90r-PC blend. This decline aligns with the DSC trends, where lower enthalpy values signify a less ordered crystalline structure. For instance, the melting enthalpy (ΔH_m_) decreased from 63.4 J/g for pure PLA to a mere 1.52 J/g in the 10PLA90r-PC blend, while cold crystallization enthalpy (ΔH_cc_) dropped to 0.56 J/g for the same composition [39].

**Figure 4:**
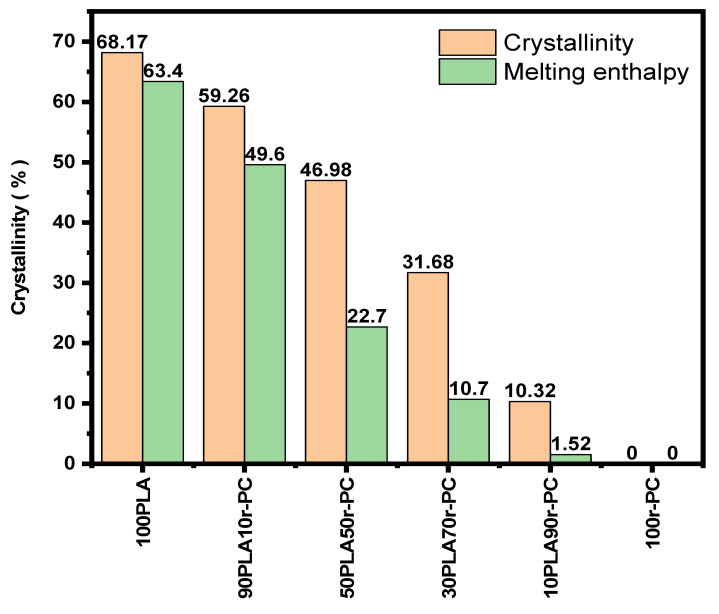
Effect of introducing r-PC into PLA blends on crystallinity at ratios of 100PLA, 90PLA10r-PC, 70PLA30r-PC, 50PLA50r-PC, 30PLA70r-PC, 10PLA90r-PC, and 100r-PC.

These observations confirm that the incorporation of r-PC disrupts the crystallization process, hindering the formation of ordered crystalline regions within the PLA matrix. The amorphous phase of r-PC acts as a barrier, inhibiting the arrangement of PLA chains and resulting in an amorphous-dominated structure. This is further validated by Figure 4, which illustrates the steady decline in crystallinity with the increase in the r-PC content [37,39].

Adding r-PC significantly alters the thermal and structural properties of PLA blends by reducing crystallinity and modifying thermal transitions. Blends with higher r-PC concentrations exhibit a more amorphous-dominated character and enhanced thermal stability, making them suitable for applications where reduced crystallinity and improved thermal resistance are desirable.

**Figure 5:**
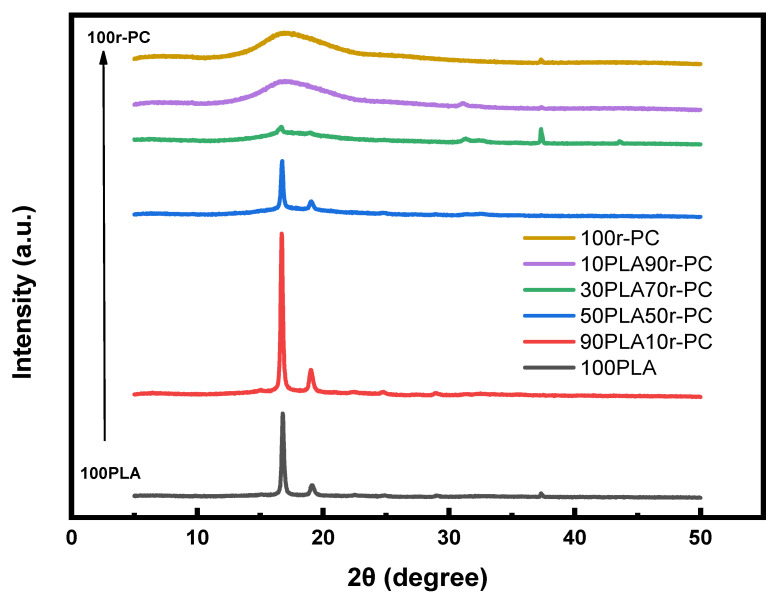
X-ray diffraction patterns of PLA/r-PC blends at ratios of 100PLA, 90PLA10r-PC, 50PLA50r-PC, 30PLA70r-PC, 10PLA90r-PC, and 100r-PC.

X-ray diffraction (XRD) was used to evaluate the crystallinity and structural changes in the PLA/r-PC blends, with results summarized in Figure 5 and Table 3. The pure PLA exhibited distinct diffraction peaks at 2θ values of approximately 16.80° and 19.14°, characteristic of its semicrystalline nature. However, as the r-PC content increased, these peaks became broader and less intense, indicating a substantial reduction in crystallinity.

The XRD data (Figure 5) show that intermediate compositions, such as 50PLA50r-PC and 30PLA70r-PC, displayed features of both crystalline and amorphous phases, suggesting partial phase compatibility. At higher r-PC concentrations, phase separation became dominant, further suppressing crystallization. These findings are consistent with previous studies on PLA-based blends, which report that adding amorphous components disrupts molecular packing, reducing crystallinity and increasing structural disorder.

Table 3 summarizes the full-width at half-maximum (FWHM) values and crystalline sizes (L) for the various PLA/r-PC blends. The crystallite size of PLA at 16.80° 2θ showed a significant reduction, dropping from 43.72 nm in the pure PLA to just 1.38 nm in the 10PLA90r-PC blend. This marked decrease highlights the disruptive effect of r-PC on PLA’s ordered crystalline domains, emphasizing the amorphous nature of r-PC. For example, in the 30PLA70r-PC blend, the reduction in peak intensity was accompanied by the appearance of a new diffuse peak, suggesting the formation of a more amorphous phase and enhanced intermixing between PLA and r-PC. The amorphous r-PC hinders the molecular alignment required for crystal growth, resulting in smaller, more defective crystallites. At lower r-PC contents, partial compatibility between PLA and r-PC allows the formation of small crystalline domains. However, as the r-PC content increases, phase separation and structural disorder become more pronounced, further reducing crystallinity [38].

The progressively increasing FWHM values across the blends point to a greater structural disorder, which aligns with the DSC findings that demonstrate a reduced crystallization ability in PLA/r-PC systems. Additionally, the broadening of XRD peaks at higher r-PC concentrations underscores the formation of new amorphous regions, further reinforcing the trend of reduced crystallinity in these blends.

### 3.3. Effect of r-PC Addition on the Tensile Toughness of PLA Blends

**Figure 6:**
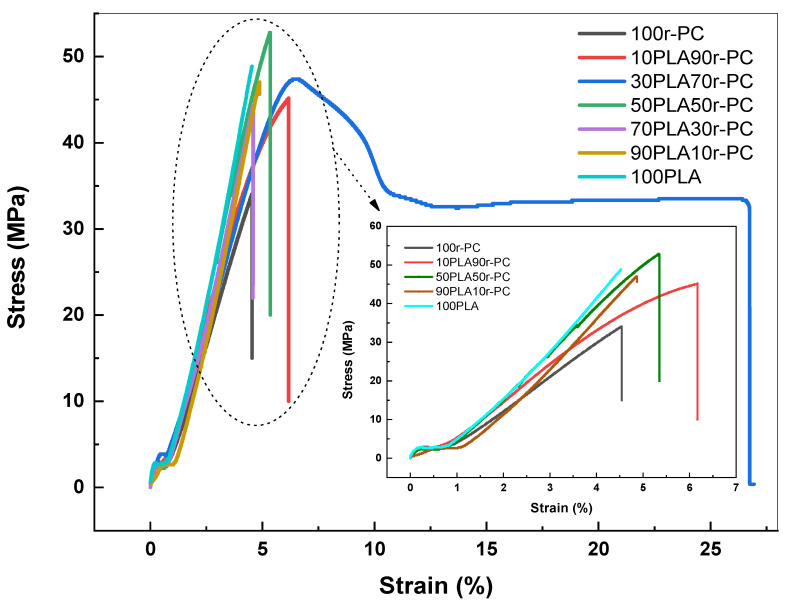
Stress–strain curves of PLA/r-PC blends at ratios of 100PLA, 90PLA10r-PC, 70PLA30r-PC, 50PLA50r-PC, 30PLA70r-PC, 10PLA90r-PC, and 100r-PC.

The tensile properties of PLA/r-PC blends were analyzed to evaluate their mechanical performance, with the results being detailed in Table 4 and illustrated in Figure 6. The pure PLA demonstrated a tensile strength of 48.8 MPa and a Young’s modulus of 1255 MPa, reflecting its inherent stiffness but limited ductility.

Blending PLA with r-PC induced significant changes in tensile toughness, with the mechanical responses varying depending on the blend composition. Notably, the 30PLA70r-PC blend exhibited the highest toughness, reaching 8725 kJ/m^3^, a remarkable tenfold increase compared to the pure PLA (924 kJ/m^3^). This improvement reflects the blend’s ability to absorb substantially more energy before failure.

The stress–strain curves in Figure 6 further validate this observation, displaying an extended strain region and enhanced energy dissipation for the 30PLA70r-PC blend. These enhancements can be attributed to improved energy absorption due to the partially effective interfacial adhesion between PLA and r-PC at this specific ratio, which facilitates stress transfer and delays fracture. However, at a higher r-PC content, the mechanical performance deteriorates, with brittleness becoming more pronounced. This can be attributed to reduced interfacial bonding, which limits the blend’s energy absorption capacity and leads to brittle failure as the secondary phase dominates the blend’s behavior. Such behavior is consistent with findings in other polymer blend systems, where the incorporation of a flexible phase dramatically enhances both impact resistance and ductility, as documented in studies on PLA blended with amorphous polymers [36,40].

In the case of PLA/r-PC blends, poor interfacial bonding at high r-PC content results in insufficient compatibility, causing premature failure under stress and a transition to brittle fracture behavior. The phase separation at a higher r-PC content exacerbates this issue by creating distinct polymer domains with limited interaction, which reduces stress transfer efficiency. This separation facilitates micro-void formation at the interface, acting as initiation sites for cracks that propagate under mechanical stress, leading to brittleness. The 30PLA70r-PC blend represents the optimal ratio in this study, providing a desirable combination of toughness and mechanical performance. These results highlight the potential for tailored PLA/r-PC blends in applications requiring improved toughness without sacrificing material integrity. Similar trends have been observed in PLA/PMMA and PBAT systems, as highlighted in previous studies [41,42]. These studies demonstrate that excessive secondary phases disrupt the material’s mechanical integrity, validating the observations in this study. Optimizing the blend composition is therefore crucial to achieving a balance between strength and flexibility [41].

**Figure 7:**
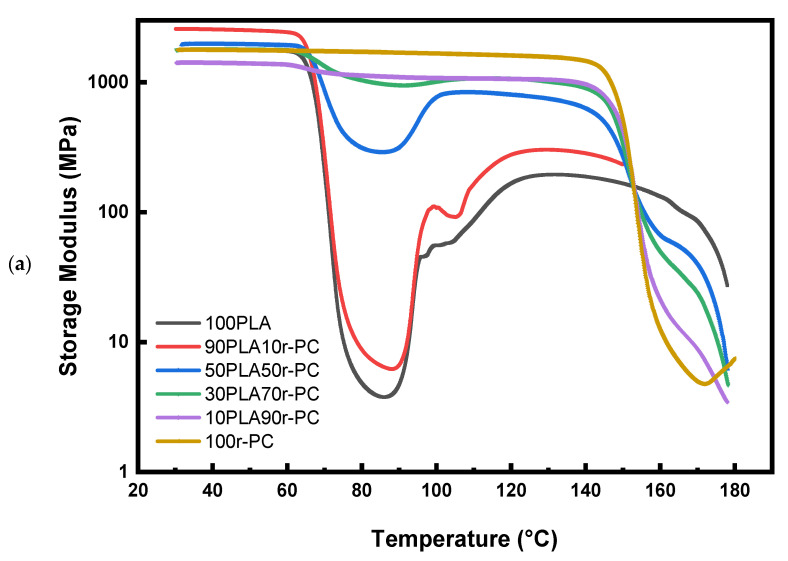

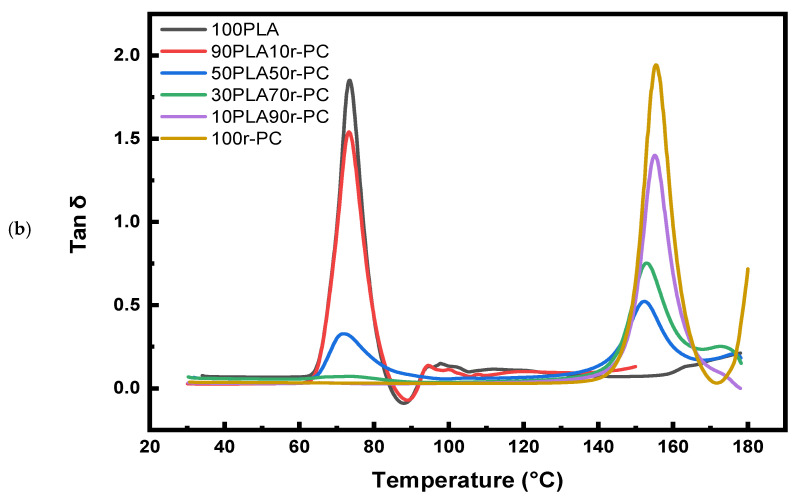
Storage modulus and tan δ curves of PLA/r-PC blends at various ratios: 100PLA, 90PLA10r-PC, 50PLA50r-PC, 30PLA70r-PC, 10PLA90r-PC, and 100r-PC. (**a**) Storage modulus vs. temperature. (**b**) Tan δ vs. temperature.

The viscoelastic properties of PLA/r-PC blends were investigated using storage modulus (E′) and tan δ curves, as shown in Figure 7. The pure PLA undergoes a sharp modulus drop around 66 °C, corresponding to its glass transition temperature (T_g_ PLA). This sudden decline highlights PLA’s poor thermal stability and brittleness, limiting its application in high-temperature environments. As the r-PC content increases, the modulus drop becomes more gradual, particularly in the 50PLA50r-PC and 30PLA70r-PC blends. This behavior is attributed to interactions between PLA and r-PC, which restrict molecular mobility, enhance stiffness at lower temperatures, and slow down modulus reduction near T_g_. Additionally, the rubbery plateau extends significantly in the 30PLA70r-PC blends, indicating improved mechanical integrity at elevated temperatures.

The tan δ curves in Figure 7b provide further insight into polymer chain dynamics and phase interactions. The pure PLA exhibits a sharp tan δ peak at approximately 61 °C, while the 30PLA70r-PC and 50PLA50r-PC blends show a shift in T_g_ along with peak broadening. This broadening T_g_ shift indicates partial miscibility as the r-PC content increases.

At ratios 50 and 70% r-PC (30PLA70r-PC and 50PLA50r-PC), the combination of a gradual modulus drops, broadened tan δ peak, and reduced crystallinity (as confirmed by XRD, Figure 5 and Table 3) indicates enhanced molecular flexibility. This flexibility facilitates localized deformation under tensile stress, which explains the observed increase in elongation at break, even though the material remains in a glassy state at room temperature. This behavior aligns with previous studies on PLA-based blends, where T_g_ shifts and peak broadening serve as indicators of partial compatibility. Furthermore, the decrease in tan δ intensity suggests stronger molecular interactions, leading to improved toughness and reduced brittleness [42].

**Figure 8:**
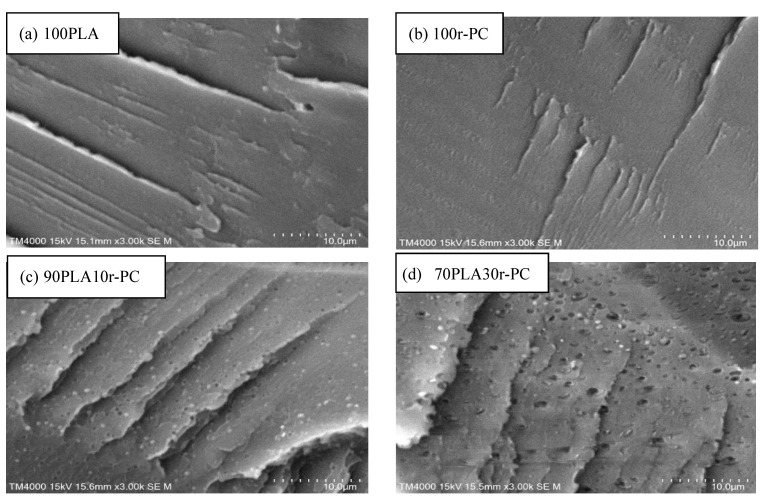

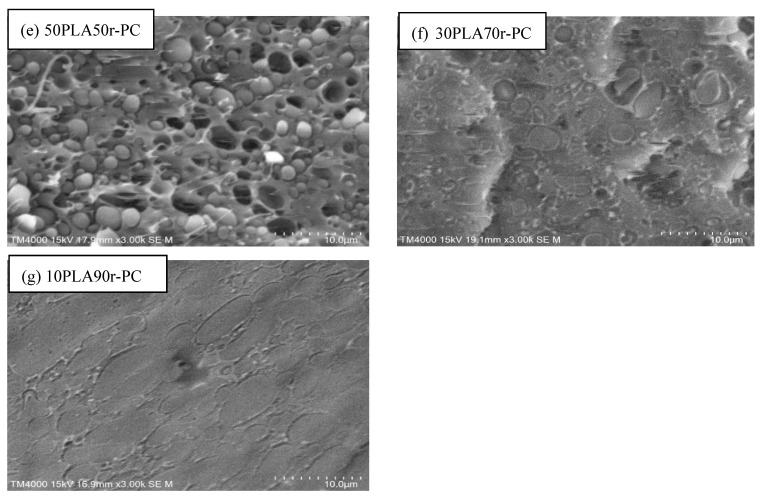
SEM micrographs of PLA/r-PC blends at various ratios: (**a**) 100PLA, (**b**) 100r-PC, (**c**) 90PLA10r-PC, (**d**) 70PLA30r-PC, (**e**) 50PLA50r-PC, (**f**) 30PLA70r-PC, and (**g**) 10PLA90r-PC.

The microstructural features of PLA/r-PC blends were examined using SEM, with the results being displayed in Figure 8. The pure PLA (Figure 8a) exhibited a smooth and homogeneous fracture surface, characteristic of brittle failure. In contrast, r-PC-rich blends, such as 30PLA70r-PC (Figure 8f), displayed a rougher and more irregular morphology.

Intermediate compositions, such as 70PLA30r-PC (Figure 8d), exhibited a partially compatible phase morphology, where PLA and r-PC coexisted with moderate interaction. However, at higher r-PC contents (e.g., 10PLA90r-PC, Figure 8g), the phase separation became more pronounced, leading to a decline in mechanical properties. The SEM micrographs of high r-PC-content blends revealed larger voids and distinct phase domains, reflecting excessive r-PC disrupting the matrix continuity. This disruption weakens stress transfer across the material, which may contribute to reduced mechanical performance. Previous studies, such as those published by Deng et al. [38], have demonstrated that poor interfacial adhesion in polymer blends significantly limits stress transfer between phases, causing premature failure. Similarly, Guo et al. [42] showed that increasing the phase separation in blends with a high secondary polymer content leads to brittle fracture due to ineffective stress dissipation. In PLA/r-PC systems, insufficient interfacial bonding at elevated r-PC concentrations facilitates crack initiation and propagation under applied stress, as evidenced by the SEM images of 10PLA90r-PC and pure r-PC.

The SEM results further correlate with XRD findings, which revealed a reduced crystallite size and disrupted crystalline continuity in blends with a higher r-PC content. While blends with lower r-PC contents (e.g., 70PLA30r-PC) exhibited relatively uniform and finely dispersed phases, high r-PC compositions showed rough phase separation and distinct amorphous domains. This morphological behavior aligns with the decreasing crystallinity observed in XRD, confirming that r-PC acts as a structural modifier, reducing PLA crystallinity and altering its crystallization mechanism.

## 4. Conclusions

This study demonstrates the integration of recycled polycarbonate r-PC into PLA to overcome key limitations of PLA, such as brittleness and low thermal stability. The incorporation of r-PC led to significant improvements in thermal stability, as evidenced by higher decomposition onset temperatures, reduced weight loss, and increased char residue. These enhancements were most pronounced in blends with a higher r-PC content, highlighting the superior thermal resistance of r-PC and its ability to delay PLA degradation. Mechanical performance was also notably enhanced, particularly in the 30PLA70r-PC blend, which achieved a tenfold increase in energy absorption compared to the pure PLA, showcasing its potential for high-performance applications. However, at very high r-PC concentrations, increased phase separation and brittleness led to a decline in toughness, underscoring the importance of composition optimization. Additionally, the introduction of r-PC disrupted PLA’s crystalline structure, resulting in a shift toward an amorphous-dominated morphology. This structural change benefits applications requiring greater flexibility, as it softens the blend and enhances segmental motion, as observed in the DMTA and tan δ results. The XRD analysis further highlights the critical role of r-PC in reducing PLA crystallinity and crystalline size, driven by phase separation, structural disorder, and interference with crystal growth. These structural modifications align with SEM observations, where higher r-PC blends exhibited pronounced phase separation and reduced interfacial adhesion, negatively impacting stress transfer and mechanical properties. In contrast, the 70PLA30r-PC showed moderate phase interaction and improved compatibility, suggesting a partially miscible system. The combination of DMTA, SEM, and XRD analyses provides a comprehensive understanding of the viscoelastic, thermal, and microstructural behavior of PLA/r-PC blends. These findings confirm that r-PC serves as an effective modifier for tailoring PLA-based materials, combining sustainability with improved thermal and mechanical performance. By carefully optimizing the r-PC content, these blends can offer a robust and eco-friendly solution for industrial applications demanding enhanced toughness, flexibility, and thermal stability.

## Figures and Tables

**Table 1:** TGA parameters for PLA/r-PC blends.

Samples	T₅ wt% (°C)	T₅₀ wt% (°C)	T_max_ PLA (°C)	T_max_r-PC (°C)	PLA Weight Loss (%)	r-PC Weight Loss(%)	Total Weight Loss (%)	Char Residue (%)
100PLA	330	370	370	-	98.66	-	98.65	1.35
90PLA10r-PC	340	380	375	-	96.93	-	96.93	3.07
70PLA30r-PC	345	410	370	500	78.75	14.15	92.88	7.12
50PLA50r-PC	350	420	375	510	65.31	23.01	88.31	11.69
30PLA70r-PC	360	450	380	515	38.63	45.10	84.61	15.39
10PLA90r-PC	370	480	385	520	18.75	62.51	81.25	18.75
100r-PC	400	520	-	520	-	75.52	75.58	24.42

**Table 2:** Thermal transition data from the first, second heating, and cooling cycles of PLA/r-PC blends.

	Samples	T_g_ (°C)	T_m_ (°C)	T_c_ (°C)	∆H_m_ (J/g)	∆H_cc_ (J/g)	X_c_ (%)
First Heating	100PLA	66	172.5	108.14	59	31	30.11
	90PLA10r-PC	67	172	106.6	56.5	29	32.85
	50PLA50r-PC	66.7, 143	171.1	102.7	25.7	12.5	28.39
	30PLA70r-PC	61, 142	171.4	103	15.0	4.0	39.43
	10PLA90r-PC	64, 144	171.2	-	1.45	-	15.59
	100r-PC	141.4	-	-	-	-	-
Second Heating	100PLA	61	172.5	108.14	63.4	-	68.17
	90PLA10r-PC	64	172	106.6	49.6	-	59.26
	50PLA50r-PC	65, 138.5	170.4	102.7	22.7	0.86	46.98
	30PLA70r-PC	55.2, 139	170	103	10.7	1.86	31.68
	10PLA90r-PC	54, 142	170	-	1.52	0.56	10.32
	100r-PC	141.2	-	-	-	-	-
The following method was used to calculate the percentage crystallinity (X_c_) of the blends:							
X_c_ (%) = (ΔH_m_ − ΔH_cc)_/(ΔH_m_^0^ × w) × 100 ΔH_m_^0^: Theoretical enthalpy of melting for 100% crystalline PLA, ΔH_m_^0^ = 93 J/g; w: Weight fraction of PLA.							

**Table 3:** FWHM and crystallite size of PLA/r-PC blends from XRD.

Samples	2Theta (deg)	FWHM (deg)	L (nm)
100PLA	16.80 19.14 24.85 37.34	0.19 0.33 0.47 0.15	43.72 25.75 17.95 58.27
90PLA10r-PC	16.71 19.04 24.75 28.99	0.19 0.31 0.37 0.40	43.95 27.04 22.76 21.28
50PLA50r-PC	16.74 19.05 24.74 28.98 32.18	0.19 0.39 0.70 0.53 9.10	42.72 21.22 12.05 16.09 0.95
30PLA70r-PC	17.56 31.69 37.34 43.59	6.03 2.63 0.15 0.21	1.39 3.28 57.83 42.32
10PLA90r-PC	17.60 31.13 37.35	6.06 0.41 0.15	1.38 21.16 55.52
100r-PC	17.63 37.33	5.92 0.14	1.42 60.84

**Table 4:** Tensile properties of r-PC/PLA blends at various ratios.

Samples	Tensile Strength (MPa)	Young’s Modulus (MPa)	Elongation at Break (%)	Toughness (kJ/m^3^)
100PLA	48.8 ± 3.5	1255 ± 4.94	4.50 ± 1.5	924 ± 150
90PLA10r-PC	47.00 ± 2.2	1224 ± 3.65	4.87 ± 3.2	908 ± 130
50PLA50r-PC	52.78 ± 2.5	941 ± 4.85	5.40 ± 3.5	1293 ± 180
30PLA70r-PC	47.39 ± 2.5	1006 ± 119.2	27.00 ± 4.5	8725 ± 350
10PLA90r-PC	45.00 ± 3.1	918 ± 2.75	6.00 ± 2.2	1477 ± 210
100r-PC	34.00 ± 2.8	879 ± 3.86	4.50 ± 2.5	698 ± 100

## Data Availability

The original contributions presented in the study are included in the article, further inquiries can be directed to the corresponding author.
